# Medicare Advantage prior-authorizations are linked to racial disparities in end-of-life Home Health use

**DOI:** 10.1093/geroni/igaf122.2007

**Published:** 2025-12-31

**Authors:** Tessa Jones, Mohamed Benyamine, Evan Bollens-lund, David Meyers, Sean Morrison, Claire Ankuda

**Affiliations:** Icahn School of Medicine at Mount Sinai, New York, New York, United States; Icahn School of Medicine at Mount Sinai, New York, New York, United States; Icahn School of Medicine at Mount Sinai, New York, New York, United States; Brown University, Providence, Rhode Island, United States; Icahn School of Medicine at Mount Sinai, New York, New York, United States; Icahn School of Medicine at Mount Sinai, New York, New York, United States

## Abstract

In Medicare Advantage (MA), Hispanic and Asian or Pacific Islander decedents are less likely to receive Medicare home health (HH) compared to their counterparts in traditional Medicare. However, the impact of MA plan features, such as requirements for referrals or prior authorizations, on these disparities is unclear. We aim to investigate what MA plan features contribute to racial and ethnic disparities in HH use at the end of life. We used a 100% Medicare cohort of adults age ≥66 who died in 2019 and were enrolled in MA in their last year of life. We first fit a linear probability model with and without a MA plan-level fixed effects to determine if racial disparities were driven by within- vs. between- plan differences. We then tested interactions between decedent race/ethnicity and two MA plan characteristics: prior authorization and referral requirements for HH. All models adjusted for individual and regional characteristics. In our sample of N = 410,351, 259,175 were in MA plans that required prior authorization and 153,439 that required referral for HH. Racial and ethnic differences were primarily driven by between-plan differences, with prior authorization requirements increasing disparities. For example, prior authorization was associated with a 1.49% (95% CI -.02 – 0.05) decrease in HH use for non-Hispanic Whites, 7.17% (95% CI .10 - .05) decrease for Hispanics; and a 3.64% (95% CI .07-.05) decrease for Asian/Pacific Islanders. Our findings suggest that prior authorizations and referrals for HH, contribute to racial and ethnic disparities in the MA program.

